# Significantly Earlier Spring Migration in Most Bird Species at the Eastern Limit of Europe

**DOI:** 10.3390/ani13193031

**Published:** 2023-09-27

**Authors:** Oleg Askeyev, Arthur Askeyev, Igor Askeyev, Tim Sparks

**Affiliations:** 1 Biomonitoring Laboratory, Institute of Problems in Ecology and Mineral Wealth, Tatarstan Academy of Sciences, Kazan 420087, Russia; parus.cyanus@rambler.ru (O.A.); art.regulus@mail.ru (A.A.); archaeozoologist@yandex.ru (I.A.); 2 Department of Zoology, Poznań University of Life Sciences, 60-625 Poznań, Poland; 3 Museum of Zoology, University of Cambridge, Cambridge CB2 3EJ, UK

**Keywords:** climate change, first-arrival date, phenology, species traits, temperature

## Abstract

**Simple Summary:**

We investigate the first arrival dates in spring of 31 migrant bird species for the period 1989–2022 in the Tatarstan Republic of Russia, towards the eastern limit of Europe. Most (28) of these species significantly advanced their migration during the study period. A number of species traits were investigated to see if these influenced the advances, but no clear relationships were identified, other than that the earliest-arriving species had advanced most. Higher spring temperatures in Tatarstan were associated with earlier arrival, but the possible influences on these birds of the temperatures at wintering grounds and on migration routes remain to be investigated.

**Abstract:**

The first arrival dates of 31 species of migrant birds in the Tatarstan Republic of Russia were monitored for the 34-year period from 1989–2022. Trends in first arrival date were evaluated using regression against the year value. Patterns in arrival data with respect to species traits (habitat, migration distance, body weight, etc.) were evaluated using redundancy analysis. Relationships between first arrival dates and Tatarstan temperatures were also evaluated using regression methods of first-arrival date on monthly mean temperatures. Almost all (28 of 31) species revealed a significantly earlier migration arrival date; however, associations between arrival patterns and species traits were equivocal. Warmer temperatures were significantly associated with earlier arrival in 26 of the 31 species, but the relationship was insufficient to explain the average 11-day advance in species. For these species and in this location only the timing and location of arrival are well recorded; the exact wintering areas and migration routes, and the timing of these phases are less well understood. When these become better known, an investigation of the influence of environmental conditions (including temperature) on departure timing and passage timing and speed is recommended.

## 1. Introduction

The climate of the Earth has changed rapidly, and the reaction of organisms to these changes has also been affected. In recent decades, changes have been reported in the spring migration of birds breeding in Eurasia and North America. These phenomena are considered a response to recent climate change [1,2,3,4,5]. There have been many studies reporting advances in spring phenology in different plant and animal species over multidecadal time periods in relation to climate parameters, for example, plant flowering in Hungary, the USA, Eastern Europe and Japan [6,7,8,9], tree leafing in Russia, Switzerland and China [8,10,11], amphibian spawning globally [12], butterflies in Sweden [13], and aphids in the UK [14], to name but a few. Cohen et al. [15] report a useful global synthesis of animal phenology. Most studies of bird migration phenology in relation to global and regional climate change have focused on trends in arrival dates; for example, [16] assembled 3827 series of arrival data but only 683 series of departure data. However, the drivers of progress in response to climate change, both intra- and intercontinentally, remain poorly understood, or opinions differ [4,17,18,19]. For example, are the climatic drivers of migration phenology related to the climate of the wintering grounds, the migration routes, or the destination (breeding) locations, or to a combination of two or more of these? Are climate effects direct or indirect, for example, are they mediated by vegetation or prey development? Are effects immediate or is there a delayed effect, for example, by selection?

For birds, climate has had impacts other than on spring migration phenology [20,21,22,23,24,25]. Changes have been apparent, for example, in breeding phenology [26,27] and on autumn migration [28,29,30,31]. Bird population sizes have also been affected [32], and distributional shifts [19,33,34,35] as a consequence of climate change have been reported.

An earlier spring migration phenology in Europe has been associated with an increase in air temperature in the east of the East European Plain [17] and other countries of the European subcontinent and Africa [36,37,38,39,40]. Generally, early arrival is often related to the migration distance; arrival dates for shorter-distance migrants have advanced more than for long-distance migrants [41]. Despite contrasting views on some aspects of migration phenology, there is currently a consensus in the scientific field that climate change does strongly affect both the phenology of bird arrivals in spring, and population dynamics [42,43].

Many changes have been noted in the Tatarstan avifauna over the past quarter-century [44]. New species have been noted for the first time, and the number of breeding species has increased by 7 to 205. Many species have increased in abundance, particularly during winter, and the number of species resident in winter has nearly doubled, to 177 species. Rapid trends in bird and other populations are of concern. It appears that we are in the initial stages of serious perturbations of fauna and flora, perhaps unique for the last two millennia. Most of these changes, at least in part, will be in response to substantial climate change.

We put forward the hypothesis that climate change affects the avian phenology of spring arrival more than internal mechanisms do. We pose three questions. The first is whether climatic perturbations really cause early arrivals of birds in Eastern Europe. The second is whether ecological traits such as body mass or habitat preferences, etc., influence trends in spring arrival. Thirdly, do closely related species differ in phenological change?

Here, we investigate long-term fluctuations (1989–2022) in first arrival dates of spring migration of 31 bird species wintering in Africa, Asia or Southern Europe. These species are common breeding birds in the eastern boundary of Europe, including the study area in Tatarstan, and the majority of them are common breeding birds throughout Europe. We examine migration phenology in relation to monthly average spring air temperatures and to species traits, to look for potential drivers of change. 

## 2. Materials and Methods

**Figure 1:**
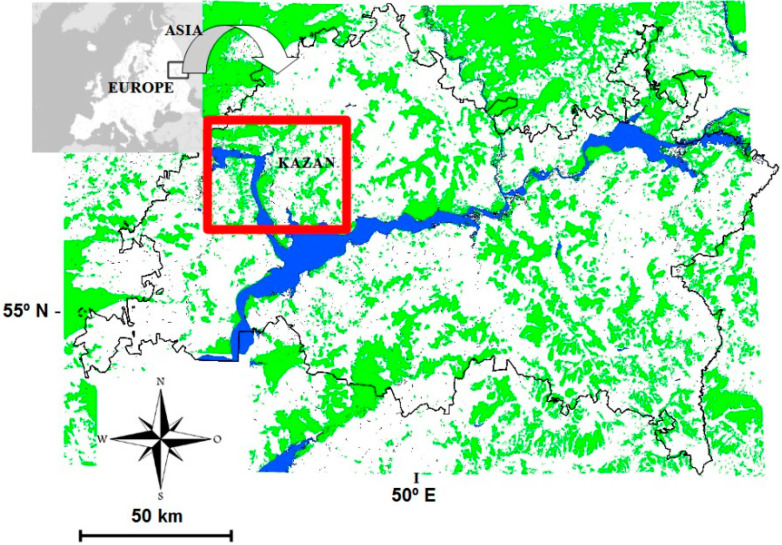
The location of Tatarstan and the city of Kazan in relation to the continents of Europe and Asia. The study area is outlined. Shading represents forests.

The Tatarstan Republic of Russia is located in the extreme east of Europe and lies between 53.58–56.40° N and 47.50–54.00° E (Figure 1). Traditionally, this area has been considered to be in the Middle Volga and PreUral region of European Russia. This region covers c. 68,000 km^2^, and includes two natural zones—forest and forest steppe—with various habitats such as sub-taiga coniferous–deciduous mixed forests, farmland, rivers, lakes and human settlements. The continental climate of Tatarstan is typical for Eastern Europe; hot summers (the average temperature of the hottest month (July) is 19 to 21 °C), and cold winters (the average temperature of the coldest month (January) is −12 to −14 °C) with annual snow cover of c.140 days.

The first observations in spring (first arrival dates) of 31 species of migrant bird were recorded each year from 1989 to 2022 on a large survey plot in Tatarstan. Habitats within the study plot include subtaiga coniferous–deciduous mixed forests, agriculture, rivers, lakes and human settlements. The study plot covers an area of c.1200 km^2^, centred on the city of Kazan (55°48′ N, 49°06′ E), and includes 5 transects totalling c.70 km on which phenological observations were made several times a week in each spring. Observations were made solely by the first three authors of this paper and the recorder effort was similar throughout the 34 years of study. The species were selected as being those which were encountered throughout the entire observation period and fully migratory (i.e., no individuals present in winter); very rare species were excluded (Table 1). Data from earlier years were excluded because they did not cover all 31 species. Dates were converted to days of the year (DOY), i.e., days after December 31, such that, for example, DOY 91 = April 1, DOY 121 = May 1. 

Monthly mean temperatures for the study area for the same years were obtained for the months of January to May. These were averages from the three met stations closest to Kazan: Opornaya (within Kazan), Raifa (30 km W of Kazan), and Laishevo (45 km SE of Kazan).

For each bird species the following traits, derived from the region where possible, were obtained from documentary sources [45,46] or based on the authors’ experience: estimates of mean body weight (g), maximum lifetime (years), population size (5-point scale derived from author’s study site data: very rare (<0.1 individuals/km^2^), rare (0.1–1 individuals/km^2^), average (>1–10 individuals/km^2^), common (>10–50 individuals/km^2^), very common (>50 individuals/km^2^)), population trend (4-point scale derived from author’s data ranging from decline to substantial increase), migration distance (intra- or intercontinental) and preferred biotypes (forest, scrub, meadows, arable, urban/garden, wet habitats) (Table 1). Trends in first arrival date (days/year) were estimated for each species from linear regression of the first arrival DOY on year. Responses of first arrival date to local temperatures were estimated for each species from multiple linear regression of the first arrival DOY on three monthly mean temperature variables, these being for the month in which mean first arrival of that species occurred and the two preceding months. Please note that no adjustment has been made here for multiple testing, and the reader should be aware that, with such a large number of tests, some significant results may have occurred by chance alone. 

Redundancy analysis (RDA) was used to compare species’ first arrival dates (34 years × 31 species) with monthly mean temperatures (34 years × 5 monthly temperatures); species data were standardised before analysis. RDA was also used to compare summaries for species’ first arrivals (mean, SD, trend, i.e., 31 species × 3 summaries) with species traits (31 species × 11 trait variables); summary variables were standardised before analysis. The relationship between trends and mean first arrival dates was investigated using Pearson correlation. Analysis was undertaken in Minitab 19 and Canoco 5.

## 3. Results

### 3.1. Trends in Arrival Dates

**Figure 2:**
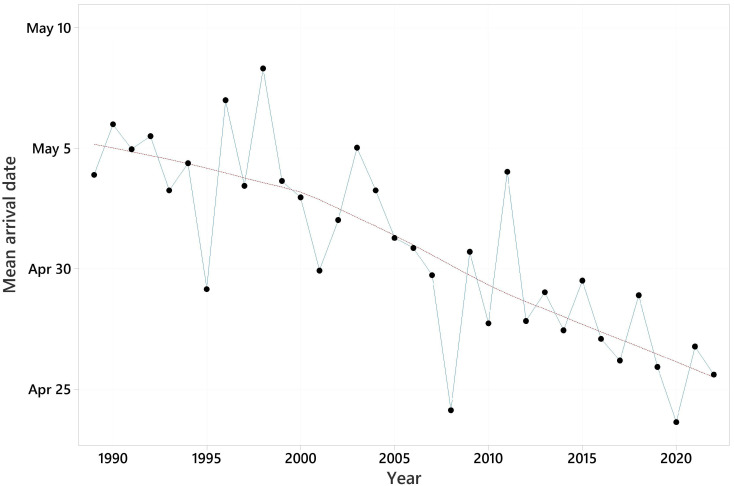
Mean first arrival date over 31 species for each of the years 1989–2022. LOWESS trend line superimposed.

Summary information on the arrival dates of the 31 species and trends in their first arrival dates are shown in Table 2. Mean first arrival dates varied from March 30, for the pied wagtail, to May 15, for Blyth’s reed warbler. All trends in first arrival date were negative, i.e., towards earlier arrival, and ranged from −0.074 days/year for the garden warbler to −0.808 days/year for the pied wagtail. These equate to 2 and 27 days earlier over the 34-year period. The mean trend was −0.310 days/year, equating to 11 days earlier over 34 years. The pattern in mean first arrival date across all species against year is shown in Figure 2. For 28 of the species, significantly earlier migration was detected. Two further species had trends with 0.05 < *p* < 0.10. 

**Table 2:** First arrival date of 31 migrant bird species in Tatarstan 1989–2022. Mean day of year (DOY), mean date, standard deviation (SD), trend (slope) and significance (p). Species are arranged in order of mean date. Significant trends are shown in bold.

	Mean				
Species	DOY	Date	SD	Slope	*p*
Pied Wagtail	89.8	Mar 30	12.01	−0.808	**<0.001**
Northern Lapwing	89.9	Mar 30	8.26	−0.552	**<0.001**
Chiffchaff	104.0	Apr 14	4.84	−0.165	**0.049**
Tree Pipit	104.5	Apr 14	5.22	−0.220	**0.013**
Bluethroat	107.5	Apr 17	5.06	−0.252	**0.003**
Pied Flycatcher	110.2	Apr 20	5.81	−0.392	**<0.001**
Willow Warbler	110.5	Apr 20	4.78	−0.285	**<0.001**
Redstart	112.6	Apr 22	6.05	−0.336	**0.001**
Barn Swallow	115.9	Apr 25	7.32	−0.480	**<0.001**
Collared Flycatcher	116.1	Apr 26	9.42	−0.747	**<0.001**
Wood Warbler	117.1	Apr 27	4.76	−0.157	0.058
Lesser Whitethroat	118.3	Apr 28	5.04	−0.261	**0.002**
Cuckoo	119.7	Apr 29	3.65	−0.183	**0.003**
Red-breasted Flycatcher	119.8	Apr 29	4.45	−0.215	**0.004**
Blackcap	121.9	May 1	5.26	−0.344	**<0.001**
Thrush Nightingale	123.1	May 3	3.72	−0.200	**0.001**
Oriental Cuckoo	125.8	May 5	3.91	−0.120	0.079
Swift	126.6	May 6	6.44	−0.484	**<0.001**
Whitethroat	128.5	May 8	5.42	−0.338	**<0.001**
Spotted Flycatcher	129.1	May 9	6.71	−0.338	**0.003**
Common Rosefinch	129.4	May 9	6.84	−0.465	**<0.001**
Greenish Warbler	130.2	May 10	4.07	−0.194	**0.005**
Garden Warbler	130.5	May 10	3.69	−0.074	0.259
Golden Oriole	130.6	May 10	5.79	−0.252	**0.010**
Corncrake	131.1	May 11	4.26	−0.240	**0.001**
Icterine Warbler	131.9	May 11	5.27	−0.332	**<0.001**
Red-backed Shrike	132.4	May 12	5.52	−0.272	**0.003**
Sedge Warbler	132.6	May 12	4.01	−0.207	**0.002**
River Warbler	135.4	May 15	4.39	−0.301	**<0.001**
Marsh Warbler	135.6	May 15	3.71	−0.173	**0.006**
Blyth’s Reed Warbler	135.7	May 15	3.59	−0.225	**<0.001**

### 3.2. First Arrival Dates and Temperature

**Figure 3:**
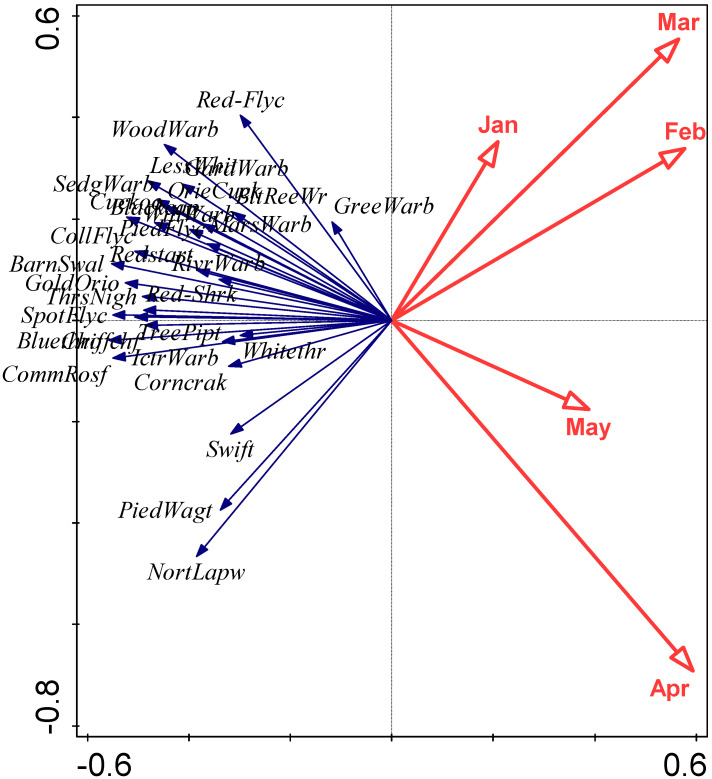
RDA biplot examining the relationships between first arrival dates and monthly mean temperatures for January to May, focussing on correlations among response variables. Eigenvalues of the first two axes are 0.19 and 0.05, respectively, and explanatory variables account for 30.8% of the total variation. Species abbreviations as follows: PiedWagt (Pied Wagtail), NortLapw (Northern Lapwing), Chiffchf (Chiffchaff), TreePipt (Tree Pipit), Bluethro (Bluethroat), PiedFlyc (Pied Flycatcher), WillWarb (Willow Warbler), Redstart (Redstart), BarnSwal (Barn Swallow), CollFlyc (Collared Flycatcher), WoodWarb (Wood Warbler), LessWhit (Lesser Whitethroat), Cuckoo (Cuckoo), Red-Flyc (Red-breasted Flycatcher), Blackcap (Blackcap), ThrsNigh (Thrush Nightingale), OrieCuck (Oriental Cuckoo), Swift (Swift), Whitethr (Whitethroat), SpotFlyc (Spotted Flycatcher), CommRosf (Common Rosefinch), GreeWarb (Greenish Warbler), GardWarb (Garden Warbler), GoldOrio (Golden Oriole), Corncrak (Corncrake), IctrWarb (Icterine Warbler), Red-Shrk (Red-backed Shrike), SedgWarb (Sedge Warbler), RivrWarb (River Warbler), MarsWarb (Marsh Warbler), BltReeWr (Blyth’s Reed Warbler).

**Figure 4:**
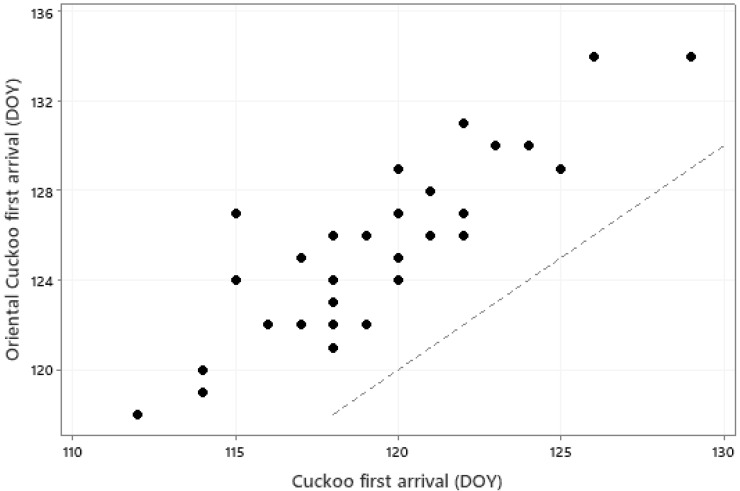
Mean first arrival date (DOY) for the Cuckoo *Cuculus canorus* and Oriental Cuckoo *Cuculus optatus* in each of the years 1989–2022 (*r* = 0.844, *p* < 0.001). The dotted line is a line of equality.

The relationships between first arrival dates of the 31 species and monthly mean temperatures of January to May are summarised in an RDA biplot in Figure 3. Species and temperatures are diametrically opposed in this biplot, confirming the negative relationships between first arrival dates and temperatures; earlier arrival is associated with higher temperatures. However, some caution in interpretation of this biplot is necessary since, for example, some species arrive before May and thus a relationship with May temperature cannot conceivably be causal. A closer examination of the influence of temperature on individual species is summarised in Table 3. Local temperatures were significantly associated (via the overall model or individual coefficients) with first arrival time in 26 of the 31 species. In each case these significant relationships were negative, implying earlier first arrival when temperatures were higher. However, the magnitude of these coefficients are not sufficient, on their own, to explain the extent of first-arrival advance observed for these species. The locations and migration routes of these birds are not fully known, so it is not possible to here consider the origin and transit temperatures, but the level of synchrony, albeit with an average 6-day offset, between first arrivals of the cuckoo *Cuculus canorus* and oriental cuckoo *Cuculus optatus* is quite remarkable, given their very different migration origins in Africa and Asia, respectively (Figure 4).

### 3.3. Relationship between Trend in First Arrival Dates and Species Traits

**Figure 5:**
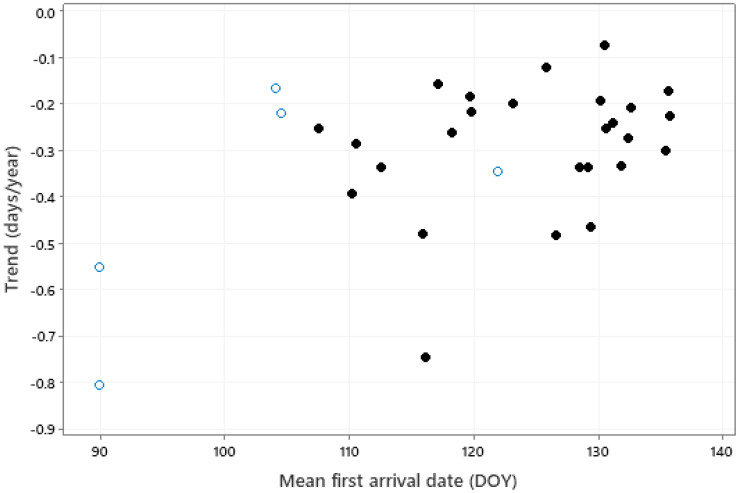
The relationship (*r* = 0.461, *p* = 0.009) between trend in first arrival date and mean arrival date (DOY, 91 = April 1, 121 = May 1). Solid symbols represent long-distance (intercontinental) migrants, open symbols represent medium-distance (intracontinental) migrants.

There was a significant correlation between the trend in first arrival and mean first arrival date (Figure 5, *r* = 0.461, *p* = 0.009); greater advances tended to be associated with bird species having earlier mean first arrival dates. No significant relationship was found between the trend in first arrival dates and any other species traits.

**Figure 6:**
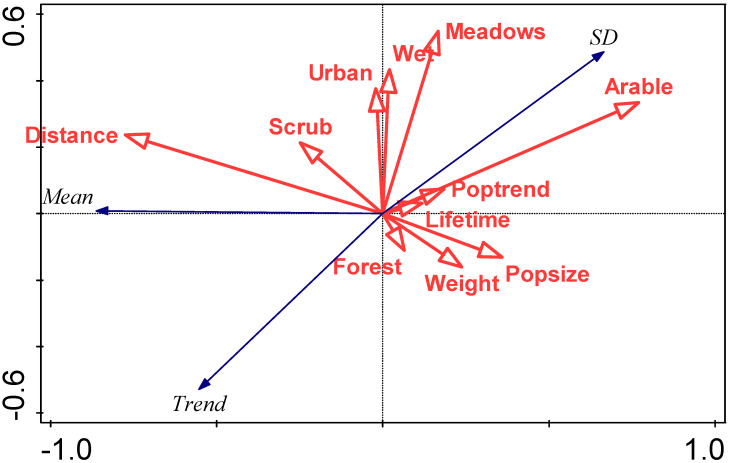
RDA biplot summarising the relationship between characteristics of the arrival distribution (mean first arrival date (mean), standard deviation in first arrival date (SD), and trend in first arrival date) and species traits, focussing on correlations among response variables. Eigenvalues of the first two axes are 0.74 and 0.01, respectively, and explanatory variables account for 74.2% of the total variation. Species traits abbreviations are: Weight (mean body weight (g)), Lifetime (maximum lifetime (years)), Popsize (population size (5-point scale)), Poptrend (population trend (4-point scale)), Distance (migration distance (0 intracontinental, 1 intercontinental)), and preferred biotopes indicated by binary variables (Forest, Scrub, Meadows, Arable, Urban (urban/garden), and Wet (wet/river)).

An RDA summarising the relationship between (i) mean first arrival dates, SD of first arrival dates, trend in first arrival date, and (ii) species traits, is summarised in Figure 6. 

## 4. Discussion

Our study shows significantly advanced spring migration phenology in 28 of 31 studied species. The significant influence of local climate could be detected in 26 species, suggesting that local climate is important in determining the timing of spring arrival, but not ruling out the influence of climate at wintering grounds or on migrations routes. A number of species traits were investigated, including body weight, migration distance, longevity, preferred habitat, population size, and population trends. With the exception of mean arrival date, none of these was significantly associated with trends in spring first arrival dates. 

The avifauna at higher latitudes consists of both resident and migratory species, and even populations of the former may contain a component of migration [47], typically short-distance intracontinental movements. At the highest latitudes, in environments of extremely cold winters, migration is the norm. 

Our studied species represent a diverse range of traits in terms of body weight, longevity, habitat preferences, migration distance, origin, and so on. Despite these fundamental differences in their ecology, almost all species showed an advance in their first arrival, with the influence of temperature apparent in most cases.

There was some evidence to suggest that advances in migration were greater for the earliest-arriving species, a feature also reported elsewhere [41]. These species tend to be those with the shorter migration distances, and for which small changes in early spring temperatures may be critical to survival. Advanced spring migration has been widely reported from cold environments [48], and from North America [30], Asia [49], and, widely, from Europe [41,43,50,51].

At present, it is believed that the beginning of spring migration is under the influence of the external environment [52], although endogenous factors were long thought to be the sole driver [53]. However, how and why species with different migration routes and wintering grounds arrive almost simultaneously in breeding areas remains poorly understood. Using only data on the timing of spring arrival it is not possible to understand all of the external influences. It would be necessary also to have reliable data on the locations of wintering grounds and migration routes, and environmental data, including temperature, for these locations. Chernetsov [54] argued convincingly of the need for data from Africa and Southern Europe to fully understand movements in Palearctic–African migrants. It has been suggested [54,55] that the speed of movement during spring migration in these species is higher, at least within Europe, than during the autumn return migration. After crossing the Mediterranean, migratory movements within Europe to breeding areas seem to take no more than 30 days [54].

More recently, studies, including those based on data from electronically tagged birds, have suggested that it is the timing of departure from Africa [56,57,58] or on migration [5,59] that determines the variability in the spring arrival of species such as those reported in the current paper. More research on the entire migration cycle of these birds is needed [60,61], and work on the consequences of asynchronous change (e.g., [62]) is gaining pace.

We have shown that arrival dates in species close in biology and ecology can occur quite synchronously (e.g., Figure 4), even if one species arrives earlier than the other and the migration origins and routes are very different. This would suggest that the temperature regime in spring in areas close to breeding sites is very influential in determining arrival dates. Synoptic maps (e.g., Plate 2.1a, Figure 7.38 in [63]) show that pan-European temperature anomalies can be quite similar. This would suggest that a migrating bird encountering warmer or colder temperatures when entering Southern Europe could generally anticipate similar anomalies at higher latitudes and adjust their stopovers, and/or migration speed, accordingly. Warming in spring across large geographical areas would thus be expected to be associated with earlier arrival at higher-latitude breeding sites. We found that species first arrived, on average, 11 days earlier over our 34-year study period, consistent with global warming. To what extent the weather conditions throughout the route determine phenological shifts in birds arriving in our region needs further examination before unequivocal statements can be made. 

Advances in bird migration arrival have been widely reported, but it is difficult to make direct comparisons between these because of differences in species, geographical location, arrival metric (for example, first date, median date), timescales, series durations, method of data collection (for example, bird observatory, citizen science, transect monitoring), and so on [43,51,64,65]. An up-to-date meta-analysis was long overdue [48], but may now have been achieved [66].

Species traits, other than perhaps earliness of arrival, did not appear to be definitively associated with trends in migration timing. Our species ranged in body weight from c.7 g for the greenish warbler to c.220 g for the northern lapwing. Similarly, species ranged in maximum lifetime expectancy from 5 to 22 years, and were representative of a wide range of population sizes, population trends, and habitat preferences. Virtually all (28 of 31) of these species exhibited a significant advance in spring migration, albeit of varying magnitudes, suggesting a major shift in ecosystem timing, regardless of species traits.

We recommend that coordinators of environmental monitoring schemes evaluate the sensitivity of their monitoring methods to potential phenological changes. As climate change continues, and possibly accelerates in the near future, there will likely be rapid changes in species phenology. We encourage future researchers to start developing additional methods that help determine the rate of phenological change and describe how these climate-driven phenological changes can be accounted for in monitoring programs. In monitoring schemes that survey several species simultaneously, coordinators need to know which species are most sensitive to climate-driven changes.

It is impossible to carry out all of the necessary research on the impact of a changing climate on bird populations solely through monitoring. We also need new breakthrough methods of laboratory research that will allow us to more accurately learn about the causes of certain changes occurring in birds, both endogenously for individuals and also exogenously for whole populations.

It should be noted that our study is based on first-arrival dates, rather than on median or mean arrival dates. The latter can only be obtained if whole populations are monitored and either marked (for example by ringing) or monitored where there are no resident breeding individuals (to avoid repeat observations). Consequently, our conclusions may not be applicable to average migration phenology, although we have no reason to suspect that average arrival dates will not also have advanced. It is unclear where many of our birds overwinter and what routes are taken for migration. Indeed, our breeding populations may not be homogenous in this respect. Consequently, it is difficult to obtain appropriate and reliable wintering and migration-route temperatures (and other climatic variables) to investigate the influences of the climates from other environments associated with the annual life cycle of these migrating animals.

## 5. Conclusions

Over a period of 34 years we show significantly earlier spring arrival in Tatarstan in 28 of 31 monitored migrant bird species. Other than the earliness of arrival, there was no obvious link between habitat, body weight, and other species traits with trends in spring-migration timing. The average advance across the 31 species was 11 days, and can be partly attributed to spring temperatures in Tatarstan, although the influence of other factors, including source and migration-route temperatures, remains to be investigated.

## Figures and Tables

**Table 1:** The 31 studied migrant bird species, their scientific names, mean body weight (g), maximum lifetime (years), population size (5-point scale), population trend (4-point scale), migration distance (0 intracontinental, 1 intercontinental), and preferred biotopes indicated by binary variables (forest, scrub, meadows, arable, urban/garden, and wet/river). Species are arranged in order of mean first arrival date, see Table 2.

Name	Scientific Name	Mean Body Weight (g)	Max Lifetime (y)	Pop Size	Pop Trend	Migration Distance	Forest	Scrub	Meadows	Arable	Urban/Garden	Wet/River
Pied Wagtail	*Motacilla alba*	22	10	5	3	0	1	1	1	1	1	1
Northern Lapwing	*Vanellus vanellus*	220	23	3	1	0	0	0	1	1	0	1
Chiffchaff	*Phylloscopus collybita*	8	6	5	4	0	1	0	0	0	0	0
Tree Pipit	*Anthus trivialis*	22	9	5	2	0	1	1	0	0	0	0
Bluethroat	*Luscinia svecica*	17	11	3	2	1	1	0	0	0	0	0
Pied Flycatcher	*Ficedula hypoleuca*	13	8	5	2	1	1	0	0	0	0	0
Willow Warbler	*Phylloscopus trochilus*	8	6	5	3	1	1	0	0	0	0	0
Redstart	*Phoenicurus phoenicurus*	15	11	5	4	1	1	0	0	0	0	0
Barn Swallow	*Hirundo rustica*	20	18	2	1	1	0	0	0	0	1	0
Collared Flycatcher	*Ficedula albicollis*	13	7	4	4	1	1	0	0	0	0	0
Wood Warbler	*Phylloscopus sibilatrix*	10	6	4	2	1	1	0	0	0	0	0
Lesser Whitethroat	*Curruca curruca*	11	8	4	3	1	1	0	0	0	1	0
Cuckoo	*Cuculus canorus*	110	19	4	4	1	1	0	0	0	0	0
Red-breasted Flycatcher	*Ficedula parva*	11	7	3	3	1	1	0	0	0	0	0
Blackcap	*Sylvia atricapilla*	17	7	4	2	0	1	0	0	0	0	0
Thrush Nightingale	*Luscinia luscinia*	21	12	4	2	1	1	0	0	0	0	0
Oriental Cuckoo	*Cuculus optatus*	102	21	2	4	1	1	0	0	0	0	0
Swift	*Apus apus*	38	16	5	4	1	1	0	0	0	1	0
Whitethroat	*Curruca communis*	15	7	5	2	1	0	1	1	0	0	0
Spotted Flycatcher	*Muscicapa striata*	15	8	5	2	1	1	0	0	0	0	0
Common Rosefinch	*Carpodacus erythrinus*	24	9	3	4	1	1	0	1	0	0	1
Greenish Warbler	*Phylloscopus trochiloides*	7	5	4	2	1	1	0	0	0	0	0
Garden Warbler	*Sylvia borin*	22	7	4	3	1	1	1	0	0	1	0
Golden Oriole	*Oriolus oriolus*	80	19	3	2	1	1	0	0	0	0	0
Corncrake	*Crex crex*	153	13	2	1	1	0	0	1	0	0	1
Icterine Warbler	*Hippolais icterina*	14	8	3	2	1	1	0	0	0	0	0
Red-backed Shrike	*Lanius collurio*	28	11	3	3	1	1	1	0	0	0	0
Sedge Warbler	*Acrocephalus schoenobaenus*	12	9	3	1	1	0	1	0	0	1	1
River Warbler	*Locustella fluviatilis*	19	10	3	1	1	1	1	1	0	0	1
Marsh Warbler	*Acrocephalus palustris*	14	11	4	1	1	0	1	0	0	1	1
Blyth’s Reed Warbler	*Acrocephalus dumetorum*	14	12	4	2	1	1	1	0	0	0	0

**Table 3:** First arrival date of 31 migrant bird species in Tatarstan 1989–2022 regressed on monthly mean temperatures for the month in which the mean first date occurs and the preceding two months. Species are arranged in order of mean date. Significant coefficients/overall models are shown in bold.

		Coefficients					
Species	Mean Date	Jan	Feb	Mar	Apr	May	*p*
Pied Wagtail	Mar 30	−0.56	−0.99	−0.77			**0.036**
Northern Lapwing	Mar 30	−0.39	0.19	**−1.89**			**0.006**
Chiffchaff	Apr 14		−0.12	−0.52	**−0.80**		**0.018**
Tree Pipit	Apr 14		−0.04	−0.71	**−0.91**		**0.013**
Bluethroat	Apr 17		−0.19	−0.56	−0.70		**0.018**
Pied Flycatcher	Apr 20		0.06	−0.24	−0.77		0.402
Willow Warbler	Apr 20		−0.29	−0.12	**−0.73**		**0.028**
Redstart	Apr 22		0.17	−0.72	**−1.04**		0.080
Barn Swallow	Apr 25		**−0.66**	+0.17	**−1.05**		**0.016**
Collared Flycatcher	Apr 26		−0.23	−0.34	−1.48		0.117
Wood Warbler	Apr 27		−0.18	−0.12	**−1.17**		**0.002**
Lesser Whitethroat	Apr 28		0.12	−0.53	**−1.22**		**0.007**
Cuckoo	Apr 29		−0.22	−0.09	**−0.64**		**0.014**
Red-breasted Flycatcher	Apr 29		0.27	−0.13	**−1.06**		**0.030**
Blackcap	May 1			−0.40	**−1.00**	−0.46	**0.026**
Thrush Nightingale	May 3			**−0.53**	**−0.69**	−0.04	**0.010**
Oriental Cuckoo	May 5			−0.36	**−0.76**	−0.19	**0.027**
Swift	May 6			−0.80	0.45	**−1.03**	**0.043**
Whitethroat	May 8			−0.28	−0.13	**−0.78**	0.228
Spotted Flycatcher	May 9			−0.67	−0.97	−0.73	**0.048**
Common Rosefinch	May 9			**−1.27**	−0.67	**−0.99**	**0.004**
Greenish Warbler	May 10			0.19	−0.25	−0.48	0.258
Garden Warbler	May 10			−0.07	**−0.62**	**−0.49**	**0.034**
Golden Oriole	May 10			−0.58	−0.58	**−1.11**	**0.011**
Corncrake	May 11			−0.36	−0.11	−0.59	0.190
Icterine Warbler	May 11			**−0.73**	−0.24	−0.40	0.171
Red-backed Shrike	May 12			−0.32	−0.66	−0.66	0.125
Sedge Warbler	May 12			−0.10	−0.53	**−0.83**	**0.006**
River Warbler	May 15			−0.26	−0.20	**−0.95**	**0.014**
Marsh Warbler	May 15			−0.24	−0.49	**−0.55**	**0.043**
Blyth’s Reed Warbler	May 15			−0.19	−0.40	**−0.52**	0.083

## Data Availability

Data will be made available on reasonable request to the lead author.

## References

[1] Gienapp P., Leimu R., Merila J. (2007). Responses to climate change in avian migration time—microevolution versus phenotypic plasticity. Clim. Res..

[2] Jonzen N., Linden A., Ergon T., Knudsen E., Vik J.O., Rubolini D., Piacentini D., Brinch C., Spina F., Karlsson L. (2006). Rapid advance of spring arrival dates in long-distance migratory birds. Science.

[3] Parmesan C. (2006). Ecological and evolutionary responses to recent climate change. Ann. Rev. Ecol. Evol. Syst..

[4] Knudsen E., Linden A., Both C., Jonzen N., Pulido F., Saino N., Sutherland W.J., Bach L.A., Coppack T., Ergon T. (2011). Challenging claims in the study of migratory birds and climate change. Biol. Rev..

[5] Haest B., Huppop O., Bairlein F. (2018). The influence of weather on avian spring migration phenology: What, where and when?. Glob. Chang. Biol..

[6] Szabo B., Vincze E., Czucz B. (2016). Flowering phenological changes in relation to climate change in Hungary. Int. J. Biometeorol..

[7] Ellwood E.R., Temple S.A., Primack R.B., Bradley N.L., Davis C.C. (2013). Record-Breaking Early Flowering in the Eastern United States. PLoS ONE.

[8] Askeyev O., Askeyev A., Askeyev I., Sparks T. (2022). Extreme temperatures help in identifying thresholds in phenological responses. Glob. Ecol. Biog..

[9] Miller-Rushing A.J., Katsuki T., Primack R.B., Ishii Y., Lee S.D., Higuchi H. (2007). Impact of global warming on a group of related species and their hybrids: Cherry tree (Rosaceae) flowering at Mt. Takao, Japan. Am. J. Bot.

[10] Meier M., Vitasse Y., Bugmann H., Bigler C. (2021). Phenological shifts induced by climate change amplify drought for broad-leaved trees at low elevations in Switzerland. Agric. For. Meteorol..

[11] Dai J., Wang H., Ge Q. (2014). The spatial pattern of leaf phenology and its response to climate change in China. Int. J. Biometeorol..

[12] While G.M., Uller T. (2014). Quo vadis amphibia? Global warming and breeding phenology in frogs, toads and salamanders. Ecography.

[13] Karlsson B. (2014). Extended season for northern butterflies. Int. J. Biometeorol..

[14] Bell J.R., Alderson L., Izera D., Kruger T., Parker S., Pickup J., Shortall C.R., Taylor M.S., Verrier P., Harrington R. (2015). Long-term phenological trends, species accumulation rates, aphid traits and climate: Five decades of change in migrating aphids. J. Anim. Ecol..

[15] Cohen J.M., Lajeunesse M.J., Rohr J.R. (2018). A global synthesis of animal phenological responses to climate change. Nat. Clim. Chang..

[16] Lehikoinen E., Sparks T.H., Moller A.P., Fiedler W., Berthold P. (2010). Changes in migration. Effects of Climate Change on Birds.

[17] Askeyev O.V., Sparks T.H., Askeyev I.V., Tishin D.V., Tryjanowski P. (2010). East versus West: Contrasts in phenological patterns?. Glob. Ecol. Biog..

[18] Charmantier A., Gienapp P. (2014). Climate change and timing of avian breeding and migration: Evolutionary versus plastic changes. Evol. Appl..

[19] Gill J.A., Alves J.A., Gunnarsson T.G. (2019). Mechanisms driving phenological and range change in migratory species. Philos. Trans. Roy. Soc. B.

[20] Møller A.P., Fiedler W., Berthold P. (2010). Effects of Climate Change on Birds.

[21] Senapathi D. (2010). Climate Change and Birds: Adaptation, Mitigation & Impacts on Avian Populations. A report on the BOU’s Annual Conference held at the University of Leicester, 6–8 April 2010. Ibis.

[22] Franks S.E., Pearce-Higgins J.W., Atkinson S., Bell J.R., Botham M.S., Brereton T.M., Harrington R., Leech D.I. (2017). The sensitivity of breeding songbirds to changes in seasonal timing is linked to population change but cannot be directly attributed to the effects of trophic asynchrony on productivity. Glob. Chang. Biol..

[23] del Mar Delgado M., Bettega C., Martens J., Päckert M. (2019). Ecotypic changes of alpine birds to climate change. Sci. Rep..

[24] Şekercioğlu Ç.H., Primack R.B., Wormworth J. (2012). The effects of climate change on tropical birds. Biol. Cons..

[25] Bateman B.L., Taylor L., Wilsey C., Wu J., LeBaron G.S., Langham G. (2020). North American birds require mitigation and adaptation to reduce vulnerability to climate change. Cons. Sci. Pract..

[26] Forchhammer M., Post E., Stenseth N. (1998). Breeding phenology and climate. Nature.

[27] McDermott M.E., DeGroote L.W. (2016). Long-term climate impacts on breeding bird phenology in Pennsylvania, USA. Glob. Chang. Biol..

[28] Van Buskirk J. (2012). Changes in the Annual Cycle of North American Raptors Associated with Recent Shifts in Migration Timing. The Auk.

[29] Haest B., Hüppop O., van de Pol M., Bairlein F. (2019). Autumn bird migration phenology: A potpourri of wind, precipitation and temperature effects. Glob. Chang. Biol..

[30] Ellwood E.R., Gallinat A., Primack R.B., Lloyd-Evans T.L., Wood E.M., Kellermann J.L. (2015). Autumn migration of North American landbirds. Phenological Synchrony and Bird Migration: Changing Climate and Seasonal Resources in North America.

[31] van Buskirk J., Mulvihill R.S., Leberman R.C. (2009). Variable shifts in spring and autumn migration phenology in North American songbirds associated with climate change. Glob. Chang. Biol..

[32] Both C., Bouwhuis S., Lessells C.M., Visser M.E. (2006). Climate change and population declines in a long-distance migratory bird. Nature.

[33] Gillings S., Balmer D.E., Fuller R.J. (2015). Directionality of recent bird distribution shifts and climate change in Great Britain. Glob. Chang. Biol..

[34] Hitch A.T., Leberg P.L. (2007). Breeding Distributions of North American Bird Species Moving North as a Result of Climate Change. Cons. Biol..

[35] Saunders S.P., Meehan T.D., Michel N.L., Bateman B.L., DeLuca W., Deppe J.L., Grand J., LeBaron G.S., Taylor L., Westerkam H. (2022). Unraveling a century of global change impacts on winter bird distributions in the eastern United States. Glob. Chang. Biol..

[36] Stervander M., Lindstrem E., Jonzen N., Andersson A. (2005). Timing of spring migration in birds: Long-term trends, North Atlantic Oscillation and the significance of different migration routes. J. Avian Biol..

[37] Kolářová E., Matiu M., Menzel A., Nekovář J., Lumpe P., Adamík P. (2017). Changes in spring arrival dates and temperature sensitivity of migratory birds over two centuries. Int. J. Biometeorol..

[38] Dolenec Z., Dolenec P. (2010). Response of the blackcap (*Sylvia atricapilla* L.) to temperature change. Pol. J. Ecol..

[39] Dolenec Z. (2022). Dates of arrival of the Eurasian golden oriole (*Oriolus oriolus* L.) in decidious forest in relation to increase of local air temperature in NW Croatia. Sumar. List.

[40] Lawrence K.B., Barlow C.R., Bensusan K., Perez C., Willis S.G. (2022). Phenological trends in the pre-and post-breeding migration of long-distance migratory birds. Glob. Chang. Biol..

[41] Rubolini D., Møller A.P., Rainio K., Lehikoinen E. (2007). Intraspecific consistency and geographic variability in temporal trends of spring migration phenology among European bird species. Clim. Res..

[42] Møller A.P., Rubolini D., Lehikoinen E. (2008). Populations of migratory bird species that did not show a phenological response to climate change are declining. Proc. Natl. Acad. Sci. USA.

[43] Sparks T.H., Bairlein F., Bojarinova J.G., Hüppop O., Lehikoinen E.A., Rainio K., Sokolov L.V., Walker D. (2005). Examining the total arrival distribution of migratory birds. Glob. Chang. Biol..

[44] Askeyev I.V., Askeyev O.V. (1999). Birds of the Tatarstan Republic.

[45] Staav R. (1998). Longevity list of birds ringed in Europe. EURING Newsl..

[46] Payevsky V.A. (2008). Demographic Structure and Population Dynamics of Songbirds.

[47] Newton I., Ekner A., Walker D., Sparks T.H. (2020). The migration seasons of birds as recorded at Dungeness Bird Observatory in southeast England. Ring Migr..

[48] Liebezeit J.R., Gurney K.E.B., Budde M., Zack S., Ward D. (2014). Phenological advancement in arctic bird species: Relative importance of snow melt and ecological factors. Polar Biol..

[49] Bozó L., Anisimov Y., Heim W. (2023). Differences in migration phenology of warblers at two stopover sites in eastern Russia suggest a longitudinal migration pattern. Avian Res..

[50] Fraixedas S., Lehikoinen A., Linden A. (2015). Impacts of climate and land-use change on wintering bird populations in Finland. J. Avian Biol..

[51] Sokolov L.V., Markovets M.Y., Shapoval A.P., Morozov Y.G. (1998). Long-term trends in the timing of spring migration of passerines on the Courish Spit of the Baltic Sea. Av. Ecol. Behav..

[52] Tsvey A., Sokolov L. (2016). Mechanisms controling the timing of spring migration in birds. Biol. Bull..

[53] Gwinner E. (1996). Circannual clocks in avian reproduction and migration. Ibis.

[54] Chernetsov N. (2012). Passerine Migration: Stopovers and Flight.

[55] Chernetsov N.S. (2016). Orientation and navigation of migrating birds. Zool. Zhurnal.

[56] Davies J.G., Kirkland M., Miller M.G.R., Pearce-Higgins J.W., Atkinson P.W., Hewson C.M. (2023). Spring arrival of the common cuckoo at breeding grounds is strongly determined by environmental conditions in tropical Africa. Proc. R. Soc. B.

[57] Ouwehand J., Both C. (2017). African departure rather than migration speed determines variation in spring arrival in pied flycatchers. J. Anim. Ecol..

[58] Bussière E.M.S., Underhill L.G., Altwegg R. (2015). Patterns of bird migration phenology in South Africa suggest northern hemisphere climate as the most consistent driver of change. Glob. Chang. Biol..

[59] Bozó L., Csörgő T., Heim W. (2021). Factors controlling the migration phenology of Siberian Phylloscopus species. J. Ornithol..

[60] Marra P.P., Cohen E.B., Loss S.R., Rutter J.E., Tonra C.M. (2015). A call for full annual cycle research in animal ecology. Biol. Lett..

[61] Zwarts L., Bijlsma R.G., van der Kamp J. (2023). The fortunes of migratory birds from Eurasia: Being on a tightrope in the Sahel. Ardea.

[62] Samplonius J.M., Atkinson A., Hassall C., Keogan K., Thackeray S.J., Assmann J.J., Burgess M.D., Johansson J., Macphie K.H., Pearce-Higgins J.W. (2021). Strengthening the evidence base for temperature-mediated phenological asynchrony and its impacts. Nat. Ecol. Evol..

[63] Blunden J., Boyer T. (2022). State of the Climate in 2021. Bull. Am. Met. Soc..

[64] Newson S.E., Moran N.J., Musgrove A.J., Pearce-Higgins J.W., Gillings S., Atkinson P.W., Miller R., Grantham M.J., Baillie S.R. (2016). Long-term changes in the migration phenology of UK breeding birds detected by large-scale citizen science recording schemes. Ibis.

[65] Horton K.G., Morris S.R., Van Doren B.M., Covino K.M. (2023). Six decades of North American bird banding records reveal plasticity in migration phenology. J. Anim. Ecol..

[66] Romano A., Garamszegi L.Z., Rubolini D., Ambrosini R. (2023). Temporal shifts in avian phenology across the circannual cycle in a rapidly changing climate: A global meta-analysis. Ecol. Monogr..

